# Wine-induced fixed drug eruption

**DOI:** 10.1016/j.jdcr.2025.11.054

**Published:** 2025-12-17

**Authors:** Jason Chen, Melissa Willis, Vincent Liu

**Affiliations:** a University of Iowa Carver College of Medicine, Iowa City, Iowa; b Department of Dermatology, University of Iowa Carver College of Medicine, Iowa City, Iowa; c Department of Pathology, University of Iowa Carver College of Medicine, Iowa City, Iowa

**Keywords:** alcohol, blistering disease, fixed drug eruption, genital dermatosis, interface dermatitis, wine

## Introduction

Fixed drug eruption (FDE) is a distinctive cutaneous drug reaction that characteristically recurs in the same location upon exposure to the offending drug.^1^ Typically, exposure to the drug requires oral ingestion of a substance, with pruritic and often painful erythematous plaques manifesting in a specific area of the body shortly after. FDE does not exhibit a known racial or sex predilection, but there may be a genetic link with human leukocyte antigen-22.^2^ Notably, the diagnosis of FDE is commonly missed due to lack of clinician awareness of this condition and its presentation being easily mistaken for more common conditions such as insect bite reactions, urticaria, or erythema multiforme.^1^ Treatment for FDE consists of discontinuation of the offending drug and symptom management through systemic antihistamines and topical corticosteroids.^1^ FDE classically presents in association with pharmacologic agents such as antibiotics, barbiturates, and non-steroidal anti-inflammatory drugs, well documented in the literature.^1^ In contrast, very few documented cases of alcohol,^3^^,^^4^ and more specifically wine,^5^ as the trigger in FDE, have been reported. Here, we report a case of alcohol-induced FDE triggered uniquely by the consumption of wine.

## Case report

A 40-year-old Caucasian male initially presented to urgent care with a blistering rash on his scrotum that had been intermittently flaring for several months. The patient had a history of psoriasis vulgaris that was well-controlled with topical medications and denied a history of infectious sexual exposure or past herpes simplex virus infection. Herpes simplex virus, gonorrhea, and chlamydia testing were negative. On exam, the rash consisted of clusters of erythematous, shallow ulcerations, with some purulent drainage. The scrotum was extremely tender, but no penile lesions were noted. Over the course of 8 health care visits, prescribed treatments included valacyclovir, trimethoprim/sulfamethoxazole, levofloxacin, clindamycin, vancomycin, probiotics, and prednisone. Computed tomography examination of the pelvis revealed no inflammatory changes nor abnormal gas or fluid collections. The patient was referred to dermatology clinic for further care.

**Fig 1:**
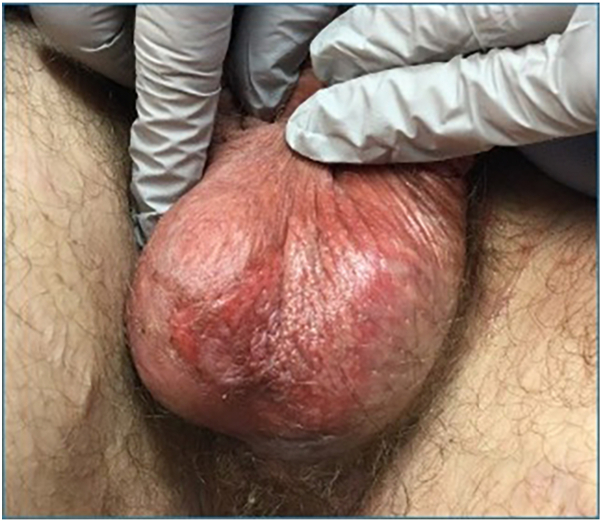
Clinical image of eruption. Desquamating peeling blister remnants with background erythema of the scrotum.

**Fig 2:**
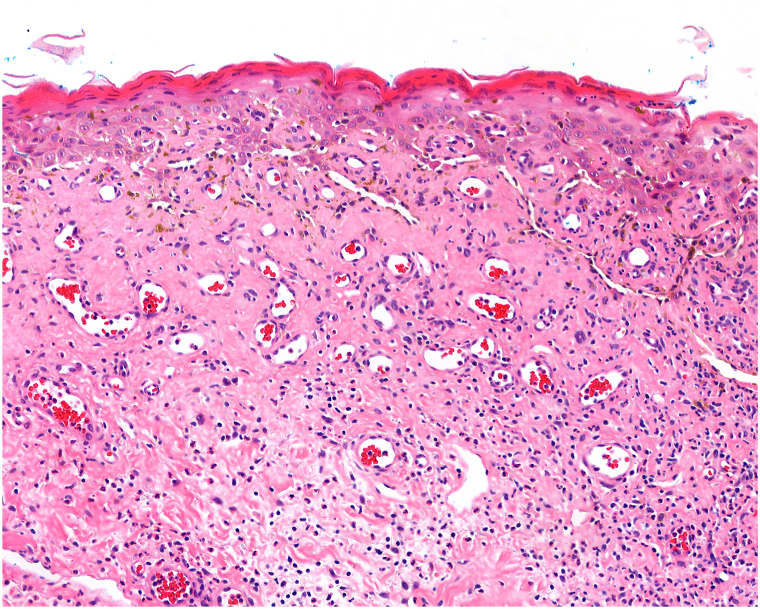
Histopathology of scrotal skin biopsy. Skin punch biopsy from the scrotum demonstrating extensive interface dermatitis with accompanying dyskeratosis and dermal melanin incontinence, associated with superficial and deep dermal perivascular lymphocytic infiltration, features compatible with fixed drug eruption [H&E; 13.6×].

Upon initial evaluation, the rash was not appreciable. The patient detailed a recurrent history of scrotal rash without an obvious trigger that resolved within days. Over 2 weeks later, the patient returned with a new flare of his scrotal rash. The rash exhibited diffuse erythema and desquamation of the scrotum with purulent drainage (Fig 1). A punch biopsy revealed features suggestive of FDE (Fig 2). Aerobic cultures revealed normal skin flora. Later that night, the patient presented to the emergency department with complaints of worsening pain and discharge from the rash after applying lidocaine ointment. A complete blood count, complete metabolic panel, urinalysis, and computed tomography of the pelvis with contrast were all unremarkable, and he was diagnosed with scrotal cellulitis. A treatment regimen consisting of clobetasol 0.05% ointment mixed 1:1 with terbinafine 1% cream, hydroxyzine, prednisone, tramadol, doxycycline, clindamycin, valacyclovir, and petroleum jelly was initiated. A week later, the patient presented to clinic with significant improvement of his rash and treatment was discontinued. Upon further directed questioning, the patient did realize that in retrospect, each cutaneous flare coincided with prior wine consumption, but no other alcohol or over-the-counter medications prior to the onset of the rash.

Two months later, the patient presented with a recurrence of his scrotal rash for the past 2-3 days with persistence despite use of clobetasol 0.05% ointment and prednisone. He reported consuming 3 glasses of wine 4 days prior, with his only other consumption of alcohol since his last visit being a single episode of beer intake. Treatment with prednisone 40 mg daily for 5 days, clobetasol 0.05% ointment twice daily for 3 days, and tacrolimus 0.1% ointment twice daily led to significant improvement and subsequent resolution of the rash in days without scarring. It was discussed that wine was the likely trigger of his FDE, and given the compelling temporal association between wine ingestion with each of the patient’s flares, patch testing was deferred.^6^ The patient was encouraged to keep a journal of alcohol consumption and treat subsequent flares with prednisone. In the 12 months since, the patient’s rash has remained quiescent, with no consumption of wine and no flares from his consumption of other types of alcohol.

## Discussion

In our patient’s case, the presentation of a recurring rash in the same groin location along with the biopsy showing interface dermatitis raised a high degree of suspicion for FDE. However, the identification of the triggering agent for this patient as a specific type of alcohol demonstrated that the pathophysiology of FDE might be far more nuanced than previously thought. Unlike previous reported cases where an instance of nonpigmenting FDE was found to be triggered by alcohol generally,^3^^,^^4^ this case further supports the idea that the mechanisms facilitating this condition can be precise enough to react to a singular type of alcohol. Currently, the only existing case of wine-induced FDE in the English literature is a case reported by Jagati *et al* which identified palm wine as the trigger,^5^ though with no explicit mention if other forms of alcohol were excluded. Furthermore, their case detailed the presence of multiple pruritic erythematous lesions over the trunk, both forearms, back, thighs, and legs in contrast to our patient who presented with a single groin lesion, thereby highlighting the varied presentations of FDE.

In summary, FDE is a condition that presents similarly to many other more common pathologies, can be triggered by a wide variety of different substances, but is generally unfamiliar to clinicians. As a result, it can often be misdiagnosed, leading to prolonged time to treatment and suboptimal patient care. This case contributes to the literature by expanding our catalog of triggers for FDE to specifically include wine and promotes awareness by clinicians of FDE as a potential diagnosis for recurrent blistering groin rash.

## Conflicts of interest

None disclosed.
